# P06-07 Steps during school hours vs throughout the day in preschoolers

**DOI:** 10.1093/eurpub/ckac095.092

**Published:** 2022-08-29

**Authors:** Susana Vale, Silvia Costa, Jorge Mota

**Affiliations:** 1 Porto, Portugal; 2 School of Sport, Exercise and Health Sciences, Loughborough University, Loughborough, United Kingdom; 3 CIAFEL-FADEUP, University of Porto, Porto, Portugal

**Keywords:** Preschool children, steps

## Abstract

**Background:**

Walking is a main form of Physical Activity (PA) and daily step counts have been used as a tool to objectively assess PA levels and patterns in many studies. Children who accumulate less than 9000 steps per day may be considered insufficiently active (Vale et al., 2015). The aim of the present study was to determine the importance of analysing all day data when evaluating PA recommendations.

**Methods:**

The study sample comprised 202 preschool aged children (44% girls), aged from 3 to 6 years (mean age of 4,7±0,8 years). Steps counts were measured during 7 consecutive days using waist worn, uniaxial Actigraph accelerometers (models 7164, 71256, and GT1M). Children used the accelerometer throughout the day, being placed after waking up and removed before going to bed. In addition to the number of steps throughout weekdays (monday to friday), the number of steps during school time was analyzed. The school hours were restricted to 8 hours and half, between 9:00h and 17:30h.

**Results:**

During all day children account 10.563 steps, 6.947 of which were recorded during school hours (p = 0.001). Looking at the entire weekday, we found that only 7% of preschool children were considered insufficiently active (9.000 steps per day). Neverthless, looking for school hours only, we found that almost half of the sample (45%) met the same recommendation. We tested for differences between all day and school day with paired t-tests.

**Conclusions:**

When looking for step counts across the entire day vs school hours, we announced that school hours, by itself, are not representative of the number of child's steps in each day. Results from this study highlight the importance of analysing all day when investigating whether preschoolers meet recommendations.

